# Variations in the Composition, Antioxidant and Antimicrobial Activities of *Cystoseira compressa* during Seasonal Growth

**DOI:** 10.3390/md20010064

**Published:** 2022-01-11

**Authors:** Martina Čagalj, Danijela Skroza, María del Carmen Razola-Díaz, Vito Verardo, Daniela Bassi, Roberta Frleta, Ivana Generalić Mekinić, Giulia Tabanelli, Vida Šimat

**Affiliations:** 1 University Department of Marine Studies, University of Split, R. Boškovića 37, HR-21000 Split, Croatia; martina.cagalj@unist.hr; 2 Department of Food Technology and Biotechnology, Faculty of Chemistry and Technology, University of Split, R. Boškovića 35, HR-21000 Split, Croatia; danci@ktf-split.hr (D.S.); gene@ktf-split.hr (I.G.M.); 3 Department of Nutrition and Food Science, Campus of Cartuja, University of Granada, 18071 Granada, Spain; carmenrazola@ugr.es (M.d.C.R.-D.); vitoverardo@ugr.es (V.V.); 4 Department for Sustainable Food Process (DISTAS), Università Cattolica del Sacro Cuore, 26100 Cremona, Italy; daniela.bassi@unicatt.it; 5 Center of Excellence for Science and Technology-Integration of Mediterranean Region (STIM), Faculty of Science, University of Split, HR-21000 Split, Croatia; roberta@stim.unist.hr; 6 Department of Agricultural and Food Sciences, University of Bologna, 40127 Bologna, Italy; giulia.tabanelli2@unibo.it

**Keywords:** *Cystoseira compressa*, microwave-assisted extraction, green extraction, biological activity, seaweed, seasonal variations, nutraceuticals, fatty acids

## Abstract

The underexplored biodiversity of seaweeds has recently drawn great attention from researchers to find the bioactive compounds that might contribute to the growth of the blue economy. In this study, we aimed to explore the effect of seasonal growth (from May to September) on the in vitro antioxidant (FRAP, DPPH, and ORAC) and antimicrobial effects (MIC and MBC) of *Cystoseira compressa* collected in the Central Adriatic Sea. Algal compounds were analyzed by UPLC-PDA-ESI-QTOF, and TPC and TTC were determined. Fatty acids, among which oleic acid, palmitoleic acid, and palmitic acid were the dominant compounds in samples. The highest TPC, TTC and FRAP were obtained for June extract, 83.4 ± 4.0 mg GAE/g, 8.8 ± 0.8 mg CE/g and 2.7 ± 0.1 mM TE, respectively. The highest ORAC value of 72.1 ± 1.2 µM TE was obtained for the August samples, and all samples showed extremely high free radical scavenging activity and DPPH inhibition (>80%). The MIC and MBC results showed the best antibacterial activity for the June, July and August samples, when sea temperature was the highest, against *Listeria monocytogenes*, *Staphylococcus aureus*, and *Salmonella enteritidis*. The results show *C. compressa* as a potential species for the industrial production of nutraceuticals or functional food ingredients.

## 1. Introduction

Among seaweeds, the brown macroalgae (Phaeophyceae) have been identified as an outstanding source of phenolic compounds, from simple phenolic acids to more complex polymers such as tannins (mainly phlorotannins). Algal phlorotannins, a group of phenolic compounds restricted to the polymers of phloroglucinol, present a heterogeneous and high molecular weight group of compounds (from 126 Da to 650 kDa) which are verified in terrestrial plants [1,2]. The phlorotannins play an important role in the cellular and ecological growth and tissue healing of alga but also show strong antioxidant, antimicrobial, cytotoxic, and antitumor properties [3,4,5,6].

Brown fucoid algae of the genus *Cystoseira sensu lato* (Sargassaceae) consist of 40 species of large marine canopy-forming macroalgae found along the Atlantic–Mediterranean coasts [7,8]. So far, a total of 214 compounds have been isolated from sixteen *Cystoseira* species, and the chemical constituents of *Cystoseira* spp. were found to contain fatty acids and derivatives, terpenoids, steroids, carbohydrates, phlorotannins, phenolic compounds, pigments and vitamins [7]. The chemical composition of the alga depends on numerous ecological factors such as temperature, salinity, UV irradiation, collecting season, depth, geographic location, thallus development, etc. However, their individual and synergistic effect on the brown alga chemical profile and biological activity is still relatively unknown. Recent studies showed that seasonality and thallus vegetative parts significantly affect the nutritional and chemical profile of alga [9]. It is considered that higher nutritional and phenolic content, higher polyunsaturated fatty acid (PUFA) content, higher vitamin and mineral content, as well as the antiproliferative properties of brown algae from brown fucoid algae were obtained during hot and dry summer seasons and higher sea temperatures [10,11,12,13]. On the other hand, no seasonal effect was recorded for the pigment profile and fucoxanthin content, nor total phenolic content and antimicrobial activity of the genera *Padina*, *Colpomenia*, *Saccharina* or *Dictyota* [14,15]. So far, there are no reports on seasonal variations in chemical profile nor the biological activity of *Cystoseira* spp.

*Cystoseira* spp. composition suggests their high nutritional value with potential applications in the nutraceutical industry. A range of 29–46% of PUFA, a low *n-6* PUFA/*n-3* PUFA ratio as well as favorable unsaturation, atherogenicity, and thrombogenicity indices were observed in several *Cystoseira* species [16]. Compounds from *Cystoseira* species are important sources of nutraceuticals and may be considered as functional foods, such as extracts of *C. tamariscifolia* and *C. nodicaulis* that were able to protect a human dopaminergic cell line from hydrogen peroxide-induced cytotoxicity and inhibit cholinesterases, while those from *C. crinita* showed significant cytotoxic activity against human breast adenocarcinoma (MCF-7 cells), inducing apoptosis and autophagy [17,18]. Besides non-volatiles, the essential oil constituents of *C. compressa* and their seasonal changes have been identified and among them for a large number of compounds a broad range of biological activities have been already proved [19]. So far, over 50 biological properties have been attributed to compounds found in genus *Cystoseira*, and the most reported are antioxidant, anti-inflammatory, cytotoxic, anticancer, cholinesterase inhibition, antidiabetic, and antiherpetic activities [7,20,21,22,23,24]. Phlorotannins are regarded as responsible for high antioxidant activity (e.g., free radical scavenging ability) [1,25,26,27]. Besides, there is little information on the antimicrobial activity of *Cystoseira* spp. extracts against major foodborne Gram-positive and Gram-negative bacteria [28]. 

The aim of this study was to investigate the chemical composition of *C. compressa*, one of the most widely distributed algae in the Adriatic Sea, to determine changes in its antioxidant and antimicrobial activity over the seasonal growth (May–September) when the algae are in the growing and reproductive phases, and the development of dense thallus occurs.

## 2. Results and Discussion

### 2.1. Total Phenolic Content, Total Tannin Content and Antioxidant Activity

Seaweed extracts were screened for total phenolic content (TPC), total tannin content (TTC) and antioxidant activity measured by ferric reducing/antioxidant power (FRAP), 2,2-diphenyl-1-picrylhydrazyl radical scavenging ability (DPPH) and the oxygen radical absorbance capacity (ORAC).

**Figure 1:**
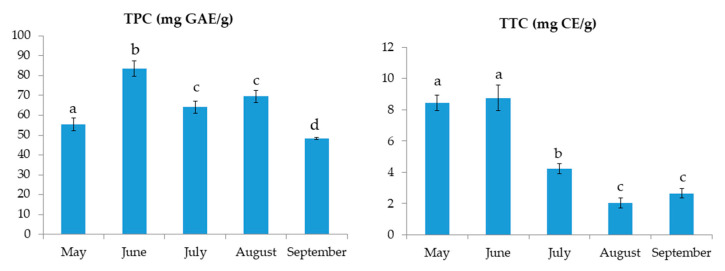
Total phenolic content (TPC) and total tannin content (TTC) of *C. compressa* extracts from May to September. ^a–d^ different letters denote statistically significant difference (*n* = 4).

**Figure 2:**
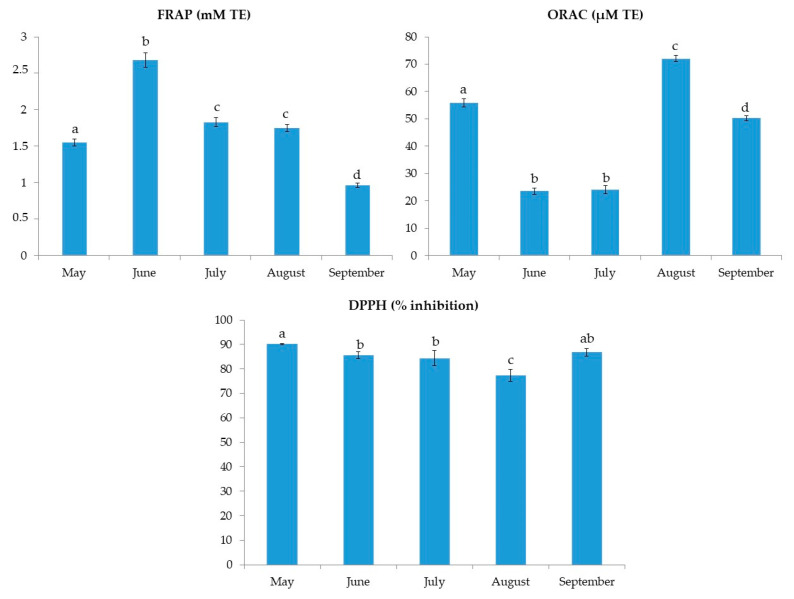
FRAP, ORAC and DPPH inhibition results for *C. compressa* extracts from May to September. ^a–d^ different letters denote statistically significant difference (*n* = 4).

The results of TPC and TTC for *C. compressa* are shown in Figure 1. The results for TPC varied from 48.2 ± 0.5 to 83.4 ± 4.0 mg GAE/g. The highest TPC was found in June samples. On the other hand, the TTC values ranged from 2.0 ± 0.3 to 8.8 ± 0.8 mg CE/g with the highest value found also in June, followed by the extract from May. The FRAP values, shown in Figure 2, ranged from 1.0 ± 0.0 to 2.7 ± 0.1 mM TE. Similar to TPC and TTC, the highest FRAP result was obtained for June, showing the reducing activity of >2.5 mM TE. TPC and FRAP results were in high correlation (0.956; *p* < 0.01). The ORAC results are shown in Figure 2. The seaweed extracts were 200-fold diluted for ORAC assay. Among the investigated samples, the highest ORAC value of 72.1 ± 1.2 µM TE was found in the August extract, with extracts from May having the second best. June and July extracts had the lowest ORAC values, more than 3-fold lower in comparison to the August extract. The DPPH radical inhibitions (in percentages) are shown in Figure 2. The extract from May had the highest inhibition (90.2%) while the August extract had the lowest inhibition (77.3%). The activity of other extracts was similar, around 85%. In the growing season, the sea temperature was the lowest in May (18.3 °C) and it rose every month till August when it peaked at 26.9 °C. Finally, a decrease in the temperature by 2.2 °C was observed in September (Table 1). 

The TPC of alga varies with seasonal changes of sea temperature, salinity, light intensity, geographical location and depth, as well as other biological factors such as age, size, the life cycle of the seaweed, presence of herbivores [1]. In this study, the geographical location and depth were eliminated as a factor as samples were collected from the same area and depth each month. The TPC, TTC and antioxidant activity results showed no correlation to the sea temperature and salinity. If the growth of alga is considered, in June when the TPC, TTC and FRAP were the highest, *C. compressa* had a fully developed, densely ramified thalli with aerocysts. In May the thalli are not yet fully developed, while in July–September it is less dense, aerocysts appear in fewer numbers [29].

The TPC of *Cystoseira* species was previously investigated and researchers reported a strong effect of harvesting location and seasonal changes, especially temperature. Mancuso et al. [12] investigated TPC in *C. compressa* from eight locations along the Italian coast and confirmed the change of TPC with geographical location. The TPC ranged between different locations from 0.1 to 0.5% of algal dry weight (DW). The authors observed the increase in TPC with the rise of sea temperature (measured at different locations). Accordingly, the highest TPC of 0.53% DW was recorded at 28 °C. In contrast, Mannino et al. [30] investigated the effect of sea temperature seasonal variation on the TPC of *C. amentacea*. They harvested algae once in every season (winter, spring, summer and autumn) and measured the sea temperature. The authors observed the highest TPC in winter (0.8% DW) when the sea temperature was the lowest. In summer and autumn, when the sea temperatures were above 20 °C, the TPCs were the lowest, 0.4 and 0.37% DW, respectively. In their study, the TPC values showed a negative correlation with sea temperature. *Cystoseira compressa* extracts, from Urla (Turkey) [31], were screened for TPC, total flavonoid content (TFC), antioxidant and antimicrobial activity. The highest TPC of 1.5 mg GAE/g and TFC of 0.8 mg QE/g were found for hexane extract while the antioxidant activity of the hexane extract measured by DPPH radical inhibition was only 21.2%, more than four-fold lower than results in our study for hydroalcoholic extracts. In comparison, the methanolic extracts (similar polarity like ethanol) showed the TPC and TFC of 0.2 mg GAE/g and 0.3 mg QE/g, respectively and two-fold lower DPPH inhibition.

Abu-Khudir et al. [18] evaluated the antioxidant, antimicrobial, and anticancer activities of cold methanolic extract, hot methanolic extract, cold aqueous extract, and hot aqueous extract from *C. crinita* and *Sargassum linearifolium*. The highest TPC was found for the cold methanolic extract of *C. crinita*, 15.0 ± 0.58 mg GAE/g of dried extract, which is more than two-fold lower than the amount detected in the September extract from our study which contained the lowest TPC. The authors also found a high content of fatty acids (44%) and their esters in *C. crinita* cold methanolic extract. Both seaweeds showed similar DPPH and ABTS radical scavenging activity with *C. crinita* cold methanolic extract having IC_50_ of 125.6 µg/mL and 254.8 µg/mL, respectively. De La Fuente et al. [32] extracted *C. amentacea* var. *stricta* with dimethyl sulfoxide (DMSO) and 50% ethanol for determining TPC, TFC and antioxidant activity of extracts by DPPH radical scavenging, FRAP, OH scavenging, and nitric oxide (NO) scavenging methods. The TPC and TFC of DMSO extracts were 65.9 µg GAE/mg and 15.8 µg QE/mg, 3.2- and 5.1-fold higher than ethanolic extracts. Similar to our results, both investigated extracts had DPPH radical scavenging activity higher than 90%. Furthermore, the DMSO extracts showed a reducing activity of almost 90% while ethanolic extract showed a higher OH radical scavenging activity. Both extracts showed very low cytotoxicity, enabling their possible use as nutraceuticals.

Oucif et al. [20] screened six seaweed species (including *C. compressa* and *C. stricta*) for TPC, DPPH radical scavenging activity and reducing power. The highest TPCs were found for *C. compressa* methanolic and ethanolic extracts, 10.24 ± 0.09 and 15.70 ± 0.72 mg GAE/g DW, respectively. *Cystoseira compressa* ethanol extract had over 90% inhibition activity for DPPH radical and the highest reducing power, which can be compared with our results. Mhadhebi et al. [24] determined TPC, DPPH and FRAP in *C. crinita*, *C. sedoides* and *C. compressa* extracts. Among the three alga, *C. compressa* extract had the highest TPC of 61.0 mg GAE/g, which is comparable to our results, the lowest DPPH IC_50_ of 12.0 µg/mL, and the highest FRAP value, 2.6 mg GAE/g.

### 2.2. Antimicrobial Activity

The results of minimal inhibitory concentration (MIC) and minimal bactericidal concentration (MBC) of the *C. compressa* extracts against common foodborne pathogens are shown in Table 2. Gram-positive bacteria were more susceptible to seaweed extracts than Gram-negative bacteria. The lowest MIC results were found against *L. monocytogenes*, for June, July, and August samples with the lowest MBC in June, and against *S. aureus* in July and August with the same MBC. There was no difference in MIC and MBC values for *E. coli* among the investigated months. June, July, and August extracts had the lowest MIC values for *S. enteritidis*. The results showed higher antimicrobial activity from June to August when the sea temperature was the highest, against all bacteria.

Alghazeer et al. [33] performed microwave-assisted extraction (MAE) on *P. pavonica* and *C. compressa*. Flavonoid-rich extracts (110.92 ± 11.38 mg rutin equivalents /g for *C. compressa*) were tested for antibacterial activity against multidrug-resistant (MDR) isolates of *S. aureus* subsp. *aureus*, *Bacillus pumilus*, *B. cereus*, *Salmonella enterica* subsp. *enteric*, and enterohemorrhagic *E. coli* using the well diffusion method, MIC and MBC. *Cystoseira compressa* extract showed stronger antibacterial activity than *P. pavonica* with inhibition zones against 14 tested isolates. The largest inhibition zones were 20.5 mm for *S. aureus* and *B. cereus*, 31 mm for *S. enterica* and 17 mm for *E. coli*. Furthermore, *C. compressa* extract had the lowest MIC (31.25 μg/mL) and MBC (62.5 μg/mL) values against *S. aureus* and *S. enterica*. Against *B. cereus*, it had an MIC value of 62.5 μg/mL and an MBC value of 125 μg/mL. The highest MIC (125 μg/mL) and MBC (500 μg/mL) values were found against *E. coli*. Maggio et al. [28] evaluated the antibacterial activity of eight brown seaweeds, six belonging to the genus *Cystoseira* (including *C. compressa*) and two belonging to the Dictyotaceae family, against *E. coli*, *Kocuria rhizophila*, *S. aureus* and a toxigenic and MDR *S. aureus* using the disk diffusion method. None of the seaweed extracts inhibited the growth of *E. coli*. *Cystoseira compressa* and *Carpodesmia amentacea* extracts showed antibacterial activity against *K. rhizophila*, *S. aureus* and MDR *S. aureus*. Abdeldjebbar et al. [34] tested the antibacterial effect of *C. compressa* and *P. pavonica* acetonic extracts against *E. coli* and *S. aureus*. The antibacterial activity was measured by disk diffusion method and MIC determination. *Cystoseira compressa* extract had 14 mm inhibition diameter for *E. coli*, showing better antibacterial activity than *P. pavonica* (12 mm). However, MIC values were not detected for *C. compressa* against both bacteria. *Padina pavonica* extract had an MIC of 50 µL for tested strains. Both extracts had a 10 mm inhibition diameter for *S. aureus*. The authors also tested the synergy of these two extracts at a 1:1 ratio. The mixture showed significant synergistic effect against *E. coli* and *S. aureus* with 16 and 12 mm inhibition diameters, respectively. The antibacterial activity of a *C. crinita* cold methanolic extract was evaluated by the disk diffusion method [18]. The extract showed the highest inhibition zones for *E. coli*, *Klebsiella pneumoniae*, *Proteus mirabilis*, *Bacillus subtilis*, *S. aureus* and *Streptococcus aureus*, with 10.5, 12.8, 10.2, 12.6, 13.3 and 11.2 mm, respectively. 

*Cystoseira compressa* extracts [31] showed moderate activity against *E. coli*, *S. aureus*, *Streptococcus epidermidis*, *E. faecalis*, *Enterobacter cloacae*, *Klebsiella pneumonie*, *B. cereus* and *P. aeruginosa*. In this study, the authors found the lowest MIC value of 32 µg/mL for both methanolic extract against *S. epidermidis* and chloroform extract against *E. cloacae*. Dulger and Dulger [21] tested *C. compressa* water and ethanol extracts against methicillin-resistant *S. aureus* (MRSA). Ethanol extract had the lowest MIC of 3.2 mg/mL and MBC of 6.3 mg/mL. 

In the above-mentioned studies, the chemical content of the investigated algae was not correlated with the antimicrobial activity, however, it is evident that *Cystoseira* spp. shows some potential to be used nutraceuticals and therapeutic purposes.

### 2.3. Chemical Analysis by UPLC-PDA-ESI-QTOF

**Figure 3:**
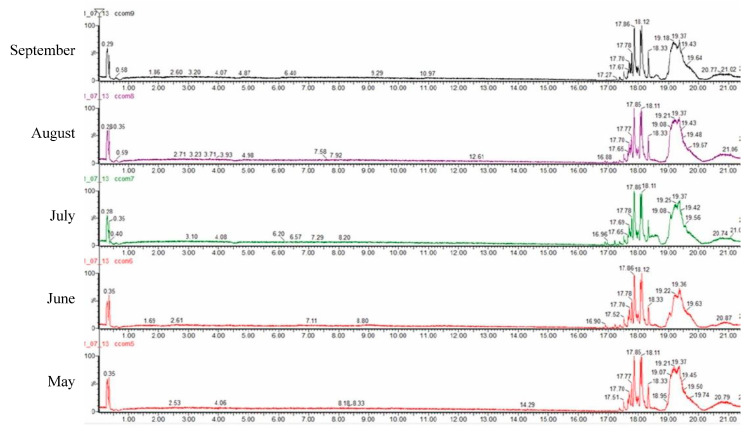
Chromatograms of the HPLC-qTOF-MS analyses of *C. compressa*.

A quali-quantitative analysis of the polar compounds from *C. compressa* extracts was achieved by LC-ESI-QTOF-MS analysis in negative ion mode. The base peak chromatograms obtained are shown in Figure 3. A total of 49 compounds were identified and the results are shown in Table 3, along with their retention time, observed and theoretical *m*/*z*, error (ppm), score (%), molecular formulae and in source fragments. In all cases, the score remained higher than 90% and the error lower than 5 ppm. All the compounds we tentatively identified according to Bouafif et al. [35] who previously found most of them in *Cystoseira* and PubChem database. Furthermore, the amount of each compound is expressed as a percentage calculated based in the areas for each extract.

The most dominant compound tentatively identified was oleic acid (C18:1n-9) with a content more than 15% in all tested samples, highest in May. The other two dominant compounds were palmitoleic acid (C16:1n-7) and palmitic acid (C16:0) also showing highest content in the May extract. Except for highly represented fatty acids, ω-3 eicosapentaenoic acid (EPA) was also found, with the highest content in July.

Low molecular weight phenolic compounds were not identified. This does not confirm that the phenolics are not present, but the main phenolics in algae are probably present as tannins (phlorotannins) that cannot be determined by HPLC-ESI-TOF-MS because they cannot be ionized due to their high molecular weight. Maggio et al. [28] reported citric acid, isocitric acid, vanillic acid methyl ester, vanillic acid sulfate, gallic acid, dihydroxybenzoic acid, 2-hydroxy-6-oxo-6-(2-hydroxyphenoxy)-hexa-2,4-dienoate, phloracetophenone, bromo-phloroglucinol, vanillylmandelic acid and exifone in *C. compressa*. The compounds were identified without quantification. Previously, vanilic acid, hydroxybenzoic acid, gallocatechin, carnosic acid, phloroglucinol, and hydroxytyrosol 4-*O*-glucoside were identified as main phenolics in fucoidan algae *Sargassum* sp. [36]. 

Jerković et al. [37] investigated fucoidal brown alga *Fucus virsoides* and found 42.28% oleic acid, 15.00% arachidonic acid and 10.51% myristic acid in its fatty acid composition. The authors used high performance liquid chromatography–high-resolution mass spectrometry (HPLC-ESI-HRMS) to determine the composition of less polar non-volatile compounds. The major compounds tentatively identified belonged to five groups, steroids, terpenoids, fatty acid glycerides, carotenoids, and chlorophyll derivatives. Fatty acid glycerides were dominant, which is comparable to our study.

Ristivojević et al. [38] identified the bioactive compounds responsible for the radical scavenging and antimicrobial activities of *Undaria pinnatifida* and *Saccharina japonica* methanolic extracts using the high-performance thin layer chromatography (HPTLC)-bioautography assay and ultra-high-performance liquid chromatography (UHPLC)-LTQ-MS/MS combined. They reported eicosapentaenoic, stearidonic and arachidonic acids as major compounds accountable for these activities. Their findings are in accordance with previous reports on PUFAs having antimicrobial activity against bacteria, viruses and fungi [39,40]. 

PUFAs, such as EPA, docosahexaenoic acid (DHA) and linolenic acid (LNA), showed in vitro antibacterial activity against *Helicobacter pylori*, *S. aureus*, Methicillin-resistant *S. aureus* (MRSA), *Vibrio vulnificus*, and *Streptococcus mutans*, inhibiting bacterial growth or altering their cell morphology [40]. To deactivate microbial cells, PUFAs directly affected the cell membranes, enhanced free radical generation, and increased the formation of cytotoxic lipid peroxides and their bioactive metabolites increasing the leukocytes’ and macrophages’ phagocytic action [39]. EPA and DHA extracts showed antimicrobial activity against foodborne pathogenic bacteria, *L. monocytogenes*, *B. subtilis*, Enterobacter aerogenes, *E. coli*, *S. aureus*, *S. enteritidis*, *S. typhimurium*, and *P. aeruginosa* [41]. The authors reported the lowest MIC value of 250 µg/mL for DHA extract against *P. aeruginosa*. A low MIC value of 350 µg/mL was found for EPA extract against *L. monocytogenes*, *B. subtilis* and *P. aeruginosa*, and for DHA extract against *L. monocytogenes* and *B. subtilis*. Besides, Cvitković et al. [42] investigated the extraction of lipid fractions from *C. compressa*, *C. barbata*, *F. virsoides*, and *Codium bursa*. In agreement with our results, the dominant fatty acids in all seaweeds were palmitic, oleic and linolenic fatty acids. *Cystoseira compressa* and *C. barbata* had the highest amounts of omega-3 EPA and DHA. *Cystoseira compressa* had 20.35% oleic acid, 17.66% arachidonic acid, 14.86% linoleic acid, 11.92% palmitic acid and 8.72% linolenic acid. Bacteria *S. aureus* can be inhibited by most free fatty acids: Lacey and Lord [43] seeded this bacterium on human skin and then applied LNA to the skin which resulted in the rapid death of the seeded bacteria. EPA (C20:5n-3) was found to successfully inhibit the growth of *S. aureus* and *B. cereus* with a 64 mg/L MIC value [44]. Oleic acid was confirmed in vitro and in vivo to effectively eliminate MRSA by disrupting its cell wall [45]. 

## 3. Materials and Methods

### 3.1. Sample Collection

*Cystoseira compressa* samples were collected off the south coast of the island Čiovo in the Adriatic Sea from May to September 2020 (43.493389° N, 16.272505° E). Sampling was done throughout a lagoon at 25 points in a depth range of 20 to 80 cm. The sea temperature and salinity were measured during sampling using a YSI Pro2030 probe (Yellow Springs, OH, USA). A sample of this species is deposited in the herbarium at the University Department of Marine Studies in Split.

### 3.2. Pre-Treatment and Extraction

Prior to the extraction, harvested algal samples were washed with tap water to remove epiphytes. Samples were then freeze-dried (FreeZone 2.5, Labconco, Kansas City, MO, USA) and ground. Based on the previous research [46] seaweeds were extracted using MAE in the advanced microwave extraction system (ETHOS X, Milestone Srl, Sorisole, Italy). Seaweeds were mixed with 50% ethanol, using 1:10 (*w*:*v*) algae to solvent ratio and extracted for 15 min at 200 W and 60 °C. The extracts were further centrifuged at 5000 rpm for 8 min at room temperature and the supernatant was filtered. The ethanolic solvent was evaporated at 50 °C and the rest of the extracts freeze dried. 

### 3.3. Determination of Total Phenolics, Total Tannins and Antioxidant Activity 

The crude algal extracts were dissolved in 50% ethanol prior to analyses in the concentration of 20 mg/mL. Folin–Ciocalteu method [47] was used for determining the TPC. Briefly, 25 μL of the extract was mixed with 1.5 mL distilled water and 125 μL Folin–Ciocalteu reagent. The solution was stirred and 375 μL 20% sodium carbonate solution and 475 μL distilled water was added after one minute. Samples were left in the dark at room temperature for 2 h. The absorbance was read using a spectrophotometer (SPECORD 200 Plus, Edition 2010, Analytik Jena AG, Jena, Germany) at 765 nm. Results were expressed as gallic acid equivalents in mg/g of freeze-dried extract (mg GAE/g).

The TTC was measured according to Zhong et al. [36] with some modifications. Briefly, 25 µL of the sample, 150 µL 4% (*w*/*v*) ethanolic vanillin solution, and 25 µL 32% sulfuric acid (diluted with ethanol) were added to the 96-well plate and mixed. The plate was incubated for 15 min at room temperature and absorbance was read at 500 nm using the microplate reader (Synergy HTX Multi-Mode Reader, BioTek Instruments, Inc., Winooski, VT, USA). The TTC results were expressed as mg catechin equivalents per g of dried extract (mg CE/g).

The reducing activity was measured as FRAP (ferric reducing/antioxidant power) [48]. Briefly, 300 μL of FRAP reagent solution was pipetted into the microplate wells, and absorbance at 592 nm was recorded. Then, 10 μL of the sample was added to the FRAP reagent and the change in absorbance after 4 min was measured. The change in absorbance, calculated as the difference between the final value of the absorbance of the reaction mixture after a certain reaction time (4 min) and the absorbance of FRAP reagent before sample addition, was compared with the values obtained for the standard solutions of Trolox. Results were expressed as micromoles of Trolox equivalents per liter of extract (μM TE).

The 2,2-diphenyl-1-picrylhydrazyl (DPPH) radical scavenging ability of extracts was also measured in 96-well microplates [49]. DPPH radical solution with the initial absorbance of 1.2 (290 μL) was pipetted into microplate wells, and absorbance was measured at 517 nm. Then, 10 μL of the sample was added to the wells and the decrease in the absorbance was measured after 1 h using the plate reader. The antioxidant activity of extracts was expressed as DPPH radical inhibition percentages (% inhibition). 

The oxygen radical absorbance capacity (ORAC) method [50,51] was performed to determine the antioxidant capacity of extracts by monitoring the inhibition of the action of free peroxyl radicals formed by the decomposition of 2,2-azobis (2-methylpropionamide)-dihydrochloride (AAPH) against the fluorescent compound fluorescein. Briefly, 150 μL of fluorescein and 25 μL of the sample in 1:200 dilution (or Trolox in the case of standard compound, or puffer in the case of blank) were pipetted into microplate wells and thermostated for 30 min at 37 °C. After 30 min, 25 μL of AAPH was added and measurements were performed at excitation and emission wavelengths of 485 and 520 nm every minute for 80 min. The results were expressed as μM of Trolox Equivalents (μM TE).

### 3.4. Determination of the Antimicrobial Activity 

The foodborne pathogens *Escherichia coli* ATCC 25922, *Salmonella enteritidis* ATCC 13076, *Enterococcus faecalis* ATCC 29212, *Listeria monocytogenes* ATCC 7644, *Staphylococcus aureus* ATCC 25923, and *Bacillus cereus* ATCC 14579 were used in this study. 

The microdilution method was used to determine the extracts’ MICs against foodborne pathogens. The extracts were dissolved in 4% DMSO (10 mg/mL) and diluted with Mueller–Hinton broth (MHB). Then, 100 µL of the mixture was added to the first well of the 96-well microtiter plate. Two-fold dilutions were done in the next wells (10–0.16 mg/mL). The 50 µL of prepared inoculum (1 × 10^5^ colony forming units (CFU)/mL determined by using the growth curves of bacteria in the *log* phase) was added to each well and plates were mixed on a microtiter plate shaker for 1 min at 600 rpm (Plate Shaker-Thermostat PST-60 HL, Biosan, Riga, Latvia). Positive control (50 µL of inoculum and 50 µL of broth media), negative control (50 µL of broth media and 50 µL of extract), blank (100 µL of broth media) and 4% DMSO were also tested. After 24 h of incubation, 20 µL of the indicator of bacterial metabolic activity, 2-*p*-iodophenyl-3-*p*-nitrophenyl-5-phenyl tetrazolium chloride (INT, in 2 mg/mL concentration) was added to each well. Plates were mixed on a plate shaker and incubated for 1 h in the dark. MIC values were read visually as the lowest concentration of the extract at which there was no detection of bacterial growth seen as the reduction of INT to red formazan [52]. 

MBC of the seaweed extracts was determined as the lowest concentration at which no microbial growth was detected on agar plates after subcultivation of bacterial suspension pipetted from wells where MIC was determined and from wells with higher extract concentrations [53].

### 3.5. Compound Analysis by UPLC-PDA-ESI-QTOF

Dried extract (3 mg) of algae was dissolved in 1 mL of MeOH/H_2_O 1/1 *v*/*v*. The analysis of compounds from algae was carried out with the use of an ACQUITY Ultra Performance LC system equipped with a photodiode array detector with a binary solvent manager (Waters Corporation, Milford, MA, USA) series with a mass detector Q/TOF micro mass spectrometer (Waters) equipped with an electrospray ionization (ESI) source operating in negative mode at the following conditions: capillary voltage, 2300 kV; source temperature, 100 °C; cone gas flow, 40 L/h; desolvation temperature, 500 °C; desolvation gas flow, 11,000 L/h; and scan range, *m*/*z* 50–1500. Separation of individual compounds was carried out using an ACQUITY UPLC BEH Shield RP18 column (1.7 μm, 2.1 mm × 100 mm; Waters Corporation, Milford, MA, USA) at 40 °C. The elution gradient test was carried out using water containing 1% acetic acid (A) and acetonitrile (B), and applied as follows: 0 min, 1% B; 2.3 min, 1% B; 4.4 min, 7% B; 8.1 min, 14% B; 12.2 min, 24% B; 16 min, 40% B; 18.3 min, 100% B, 21 min, 100% B; 22.4 min, 1% B; 25 min, 1% B. The sample volume injected was 2 μL and the flow rate used was 0.6 mL/min. The compounds were monitored at 280 nm. Integration and data elaboration were performed using MassLynx 4.1 software (Waters Corporation, Milford, MA, USA) [54].

### 3.6. Statistical Analyses

The results of antioxidant analyses were expressed as mean ± standard deviation and antimicrobial results as a mean of 3 replicas. Analysis of variance (one-way ANOVA) was used to assess the difference between TPC, TTC and antioxidant assays, followed by a least significance difference test at 95% confidence level to evaluate differences between sets of mean values [55]. Pearson’s correlation coefficient was used to determine the relation between the variables. Analyses were carried out using Statgraphics Centurion-Ver.16.1.11 (StatPoint Technologies, Inc., Warrenton, VA, USA).

## 4. Conclusions

The results obtained for the brown fucoidal macroalgae *C. compressa* from the Adriatic Sea indicated that it was a good source of compounds. The TPC and TTC content reflected a variation over the growing season, with the highest values in June. The detected FRAP showed high correlation with TPC and TTC content. The DPPH values were >80% inhibition over the whole sampling period, while the highest antioxidant activity with regards to ORAC was in August when the sea temperature was the highest. No evident correlation existed between the temperature and salinity change and TPC, TTC or antioxidant activity. From June to August, higher antimicrobial activity against foodborne pathogens was observed, especially against *L. monocytogenes*, *S. aureus* and *S. enteritidis*. Further investigations are needed to gain insight into the effect of abiotic factors, growth and thallus development of the alga on its biological potential and to discover the compounds responsible for the different biological activities.

## Figures and Tables

**Table 1:** Sea temperature and salinity recorded during the harvest of the algal samples.

	May	June	July	August	September
Temperature (°C)	18.3	21.8	22.4	26.9	24.7
Salinity (PSU)	37.4	38.1	38.3	38.3	38.3

**Table 2:** Results of the minimal inhibitory concentration (MIC, mg/mL) and minimal bactericidal concentration (MBC, mg/mL) of the seaweed extracts against foodborne pathogens (*n* = 3).

	May		June		July		August		September	
	MIC	MBC	MIC	MBC	MIC	MBC	MIC	MBC	MIC	MBC
*Escherichia coli*	10	10	10	10	10	10	10	10	10	>10
*Salmonella enteritidis*	10	10	5	10	5	10	5	10	10	>10
*Enterococcus faecalis*	10	10	10	10	10	10	5	10	10	10
*Listeria monocytogenes*	10	>10	2.5	2.5	2.5	5	2.5	5	5	>10
*Staphylococcus aureus*	10	>10	5	5	2.5	2.5	2.5	2.5	10	10
*Bacillus cereus*	>10	n.d.	10	>10	10	>10	10	>10	>10	n.d.

n.d.—not determined

**Table 3:** The compounds detected in investigated *C. compressa* samples analyzed by UPLC-PDA-ESI-QTOF.

N°	RT (min)	Observed (*m*/*z*)	Theorical (*m*/*z*)	Error (ppm)	Score (%)	Molecular Formula	In Source Fragments	Tentative Compound	May (%)	June (%)	July (%)	August (%)	September (%)
1	0.28	343.0367	343.0368	−0.3	94.18	C_20_H_4_N_6_O	-	1a,9b-Dihydrophenanthro [9,10-b]oxirene-2,3,4,7,8,9-hexacarbonitrile	4.76	4.50	4.54	4.96	6.51
2	0.29	201.0244	201.0247	−1.5	98.89	C_4_H_10_O_9_	-	2-(1,2,2,2-Tetrahydroxyethoxy)ethane-1,1,1,2-tetrol	6.67	6.17	6.58	6.72	7.90
3	0.32	141.0162	141.0161	0.7	91.01	C_2_H_2_N_6_O_2_	-	Diazidoacetic acid	1.97	2.02	2.40	2.01	1.74
4	0.35	181.0707	181.0712	−2.8	100	C_6_H_14_O_6_	101.0230; 89.0227; 71.0137; 59.0121	d-Sorbitol	3.11	3.57	1.34	2.34	1.83
5	0.40	317.0506	317.0509	−0.9	90.44	C_12_H_14_O_10_	209.0890	d-glucaric acid derivate	1.13	0.88	0.57	0.67	0.80
6	0.42	384.1510	384.1519	−2.3	92.29	C_15_H_23_N_5_O_7_	-	Threonyl-histidyl-glutamic acid	0.09	0.11	0.06	0.07	0.10
7	16.56	287.2211	287.2222	−3.8	95.91	C_16_H_32_O_4_	-	10,11-Dihydroxy-9,12-dioxooctadecanoic acid	0.19	0.19	0.20	0.17	0.20
8	16.90	275.1999	275.2011	−4.4	96.48	C_18_H_28_O_2_	231.2098; 253.0915	Stearidonic acid (C18:4n-3) isomer a	0.12	0.42	0.49	0.32	0.06
9	16.97	275.2007	275.2011	−1.5	97.68	C_18_H_28_O_2_	231.2092; 177.0854; 255.2322;	Stearidonic acid (C18:4n-3) isomer b	0.21	0.28	0.56	0.32	0.08
10	16.97	293.2112	293.2117	−1.7	92.64	C_18_H_30_O_3_	249.1835; 275.1652	13-ketooctadecadienoic acid isomer a	0.07	0.09	0.24	0.31	0.13
11	17.08	287.2211	287.2222	−3.8	95.91	C_16_H_32_O_4_	271.2083; 253.2157	10,16-Dihydroxyhexadecanoic acid isomer a	0.01	0.01	0.02	0.03	0.03
12	17.13	309.2056	309.2066	−3.2	96.09	C_18_H_30_O_4_	279.2287	6,9-Octadecadienedioic acid	0.02	0.04	0.01	0.08	0.00
13	17.18	295.2276	295.2273	1.0	100	C_18_H_32_O_3_	279.2300; 275.2019; 255.2325	9,10-Epoxyoctadecenoic acid (vernolic acid)	0.02	0.13	0.07	0.13	0.08
14	17.20	277.2159	277.2168	−3.2	91.36	C_18_H_30_O_2_	255.2321; 239.2030; 227.2013	gamma-Linolenic acid isomer a (C18:3n-6)	0.10	0.22	0.29	0.22	0.08
15	17.22	429.3009	429.3005	0.9	91.64	C_27_H_42_O_4_	273.1859; 135.0447	24-Keto-1,25-dihydroxyvitamin D3	0.01	0.57	0.58	0.05	0.02
16	17.26	247.1689	247.1698	−3.6	94.96	C_16_H_24_O_2_	233.0985	2,4,6-Triisopropyl benzoic acid	0.05	0.02	0.02	0.24	0.26
17	17.35	287.2212	287.2222	−3.5	90.62	C_16_H_32_O_4_	271.2082; 253.2158	10,16-Dihydroxyhexadecanoic acid isomer b	0.01	0.01	0.13	0.15	0.09
18	17.37	199.1694	199.1698	−2.0	90.11	C_12_H_24_O_2_	181.1062; 155.0336	Lauric acid	0.90	0.85	0.92	0.81	0.83
19	17.38	297.2426	297.2430	−1.3	98.84	C_18_H_34_O_3_	279.2367; 255.2334	10-Oxooctadecanoic acid isomer a	0.35	0.39	0.34	0.37	0.40
20	17.40	243.1952	243.1960	−3.3	90.78	C_14_H_28_O_3_	197.1907	3-hydroxymyristic acid	0.08	0.07	0.07	0.10	0.09
21	17.42	293.2112	293.2117	−1.7	94.2	C_18_H_30_O_3_	249.1833; 275.1649	13-ketooctadecadienoic acid isomer b	0.04	0.10	0.11	0.29	0.04
22	17.43	427.2827	427.2848	−4.9	90.28	C_27_H_40_O_4_	271.1716; 188.0842; 135.0441	Hydroxyprogesterone caproate	0.00	0.17	0.24	0.02	0.01
23	17.46	429.3009	429.3005	0.9	91.64	C_27_H_42_O_4_	273.1843; 135.0445	24-Keto-1,25-dihydroxyvitamin D3 isomer b	0.00	0.04	0.08	0.01	n.d.
24	17.48	295.2262	295.2273	−3.7	94.13	C_18_H_32_O_3_	279.2295; 275.2023; 255.2321	9,10-Epoxyoctadecenoic acid isomer b (vernolic acid)	0.34	0.43	0.39	0.40	0.41
25	17.51	269.2110	269.2117	−2.6	98.63	C_16_H_30_O_3_	251.2336	3-Oxohexadecanoic acid	0.07	0.16	0.01	0.26	0.11
26	17.51	225.1857	225.1855	−0.9	95.99	C_14_H_26_O_2_	188.0832; 213.1870; 175.0757	Myristoleic acid	2.35	2.14	2.26	2.15	2.22
27	17.53	255.2319	255.2324	−2.0	91.41	C_16_H_32_O_2_	225.1861; 213.1845	Hexadecanoic acid (palmitic acid) isomer a (C16:0)	0.10	0.05	0.04	0.03	0.04
28	17.57	275.2007	275.2011	−1.5	97.68	C_18_H_28_O_2_	231.2093; 255.2326	Stearidonic acid (C18:4n-3) isomer c	0.25	0.35	0.67	0.69	0.33
29	17.58	277.2152	277.2168	−5.8	99.51	C_18_H_30_O_2_	255.2289; 239.2001; 227.1989	gamma-Linolenic acid isomer b (C18:3n-6)	0.00	0.01	0.01	0.01	0.00
30	17.59	213.18458	213.1855	−3.6	92.41	C_13_H_26_O_2_	-	Tridecanoic acid	1.20	1.19	1.15	1.12	1.15
31	17.62	427.2839	427.2848	−2.1	96.07	C_27_H_40_O_4_	271.1659; 188.0827; 135.0442	Hydroxyprogesterone caproate isomer b	n.d.	0.13	0.21	0.02	0.01
32	17.62	257.2108	257.2117	−3.5	95.16	C_15_H_30_O_3_	227.2037; 211.2072	11-Hydroxypentadecanoic acid	0.10	0.09	0.09	0.07	0.11
33	17.63	251.2010	251.2011	−0.4	100	C_16_H_28_O_2_	233.9910; 207.0983	7,10-hexadecadienoic acid	0.77	0.84	0.80	0.80	0.91
34	17.64	297.2429	297.2430	−0.3	97.33	C_18_H_34_O_3_	279.2364; 255.2332	10-Oxooctadecanoic acid isomer b	0.56	0.63	0.58	0.53	0.56
35	17.66	239.2001	239.2011	−4.2	97.7	C_15_H_28_O_2_	227.2002; 159.8926	Myristoleic acid methyl ester	5.22	5.23	5.00	4.89	4.92
36	17.74	277.2162	277.2168	−2.2	99.51	C_18_H_30_O_2_	255.2318; 239.1991; 227.2015	gamma-Linolenic acid isomer c (C18:3n-6)	1.08	1.41	1.92	2.47	2.17
37	17.71	301.2158	301.2168	−3.3	99.56	C_20_H_30_O_2_	283.2283; 275.1972	Eicosapentanoic acid isomer a (C20:5n-3)	0.65	0.65	1.09	0.81	0.75
38	17.73	301.2156	301.2168	−4.0	98.12	C_20_H_30_O_2_	283.2287; 275.1957	Eicosapentanoic acid isomer b (C20:5n-3)	0.62	0.63	1.06	0.80	0.74
39	17.77	227.2001	227.2011	−4.4	93.6	C_14_H_28_O_2_	-	Tetradecanoic acid (C14:0)	5.04	5.19	5.34	5.17	5.22
40	17.81	271.2266	271.2273	−2.6	97.75	C_16_H_32_O_3_	253.0954; 225.2211	Hydroxy-palmitic acid	0.47	0.41	0.45	0.56	0.66
41	17.85	253.2156	253.2168	−4.7	96.47	C_16_H_30_O_2_	-	Palmitoleic acid isomer a (C16:1n-7)	12.65	11.93	11.45	11.57	11.74
42	17.94	241.2170	241.2168	0.8	100	C_15_H_30_O_2_	223.2081	Pentadecanoic acid (C15:0)	3.68	3.89	3.87	3.72	3.67
43	17.97	279.2314	279.2324	−3.6	98.25	C_18_H_32_O_2_	267.2340; 275.2037	Octadeca-10,12-dienoic acid (C18:2n-6)	1.07	1.14	1.29	1.31	1.27
44	18.01	267.2318	267.2324	−2.2	99.96	C_17_H_32_O_2_	249.0437; 223.0291	9-Heptadecenoic acid (C17:1n-8)	3.73	3.99	3.67	3.78	3.60
45	18.08	255.2321	255.2324	−1.2	99.9	C_16_H_32_O_2_	227.2015	Hexadecanoic acid (palmitic acid)(C16:0)	10.46	10.46	10.24	9.92	9.83
46	18.12	281.2486	281.2481	1.8	96.88	C_18_H_34_O_2_	-	Oleic acid (C18:1n-9)	15.87	15.06	15.39	15.33	15.12
47	18.22	269.2476	269.2481	−5.6	99.96	C_17_H_34_O_2_	255.2325	Heptadecanoic acid (C17:0)	5.30	5.23	5.18	5.08	4.95
48	18.33	283.2618	283.2637	−1.9	99.21	C_18_H_36_O_2_	-	Octadecanoic acid (stearic acid) C18:0	5.44	4.92	5.01	5.11	5.24
49	18.54	311.2944	311.2950	−2.0	90.87	C_20_H_40_O_2_	255.2307; 225.0060	Arachidic acid	0.68	0.62	0.67	0.66	0.68

## Data Availability

Data available on request.

## References

[1] Generalić Mekinić I., Skroza D., Šimat V., Hamed I., Čagalj M., Perković Z.P. (2019). Phenolic Content of Brown Algae (Pheophyceae) Species: Extraction, Identification, and Quantification. Biomolecules.

[2] Mannino A.M., Micheli C. (2020). Ecological Function of Phenolic Compounds from Mediterranean Fucoid Algae and Seagrasses: An Overview on the Genus Cystoseira Sensu Lato and Posidonia Oceanica (L.) Delile. J. Mar. Sci. Eng..

[3] Stiger-Pouvreau V., Jégou C., Cérantola S., Guérard F., Le Lann K., Bourgougnon N. (2014). Phlorotannins in Sargassaceae Species from Brittany (France). Advances in Botanical Research.

[4] Messina C., Renda G., Laudicella V., Trepos R., Fauchon M., Hellio C., Santulli A. (2019). From Ecology to Biotechnology, Study of the Defense Strategies of Algae and Halophytes (from Trapani Saltworks, NW Sicily) with a Focus on Antioxidants and Antimicrobial Properties. Int. J. Mol. Sci..

[5] Abdelhamid A., Jouini M., Bel Haj Amor H., Mzoughi Z., Dridi M., Ben Said R., Bouraoui A. (2018). Phytochemical Analysis and Evaluation of the Antioxidant, Anti-Inflammatory, and Antinociceptive Potential of Phlorotannin-Rich Fractions from Three Mediterranean Brown Seaweeds. Mar. Biotechnol..

[6] Šimat V., Elabed N., Kulawik P., Ceylan Z., Jamroz E., Yazgan H., Čagalj M., Regenstein J.M., Özogul F. (2020). Recent Advances in Marine-Based Nutraceuticals and Their Health Benefits. Mar. Drugs.

[7] Bruno de Sousa C., Gangadhar K.N., Macridachis J., Pavão M., Morais T.R., Campino L., Varela J., Lago J.H.G. (2017). Cystoseira Algae (Fucaceae): Update on Their Chemical Entities and Biological Activities. Tetrahedron Asymmetry.

[8] Orellana S., Hernández M., Sansón M. (2019). Diversity of Cystoseira Sensu Lato (Fucales, Phaeophyceae) in the Eastern Atlantic and Mediterranean Based on Morphological and DNA Evidence, Including Carpodesmia Gen. Emend. and Treptacantha Gen. Emend. Eur. J. Phycol..

[9] Kumar S., Sahoo D., Levine I. (2015). Assessment of Nutritional Value in a Brown Seaweed Sargassum Wightii and Their Seasonal Variations. Algal Res..

[10] Praiboon J., Palakas S., Noiraksa T., Miyashita K. (2018). Seasonal Variation in Nutritional Composition and Anti-Proliferative Activity of Brown Seaweed, Sargassum Oligocystum. J. Appl. Phycol..

[11] Gosch B.J., Paul N.A., de Nys R., Magnusson M. (2015). Seasonal and Within-Plant Variation in Fatty Acid Content and Composition in the Brown Seaweed Spatoglossum Macrodontum (Dictyotales, Phaeophyceae). J. Appl. Phycol..

[12] Mancuso F.P., Messina C.M., Santulli A., Laudicella V.A., Giommi C., Sarà G., Airoldi L. (2019). Influence of Ambient Temperature on the Photosynthetic Activity and Phenolic Content of the Intertidal Cystoseira Compressa along the Italian Coastline. J. Appl. Phycol..

[13] Britton D., Schmid M., Revill A.T., Virtue P., Nichols P.D., Hurd C.L., Mundy C.N. (2021). Seasonal and Site-Specific Variation in the Nutritional Quality of Temperate Seaweed Assemblages: Implications for Grazing Invertebrates and the Commercial Exploitation of Seaweeds. J. Appl. Phycol..

[14] Karkhaneh Yousefi M., Seyed Hashtroudi M., Mashinchian Moradi A., Ghassempour A.R. (2020). Seasonal Variation of Fucoxanthin Content in Four Species of Brown Seaweeds from Qeshm Island, Persian Gulf and Evaluation of Their Antibacterial and Antioxidant Activities. Iran. J. Fish. Sci..

[15] Marinho G.S., Sørensen A.D.M., Safafar H., Pedersen A.H., Holdt S.L. (2019). Antioxidant Content and Activity of the Seaweed Saccharina Latissima: A Seasonal Perspective. J. Appl. Phycol..

[16] Vizetto-Duarte C., Pereira H., De Sousa C.B., Rauter A.P., Albericio F., Custódio L., Barreira L., Varela J. (2015). Fatty Acid Profile of Different Species of Algae of the Cystoseira Genus: A Nutraceutical Perspective. Nat. Prod. Res..

[17] Custódio L., Silvestre L., Rocha M.I., Rodrigues M.J., Vizetto-Duarte C., Pereira H., Barreira L., Varela J. (2016). Methanol Extracts from Cystoseira Tamariscifolia and Cystoseira Nodicaulis Are Able to Inhibit Cholinesterases and Protect a Human Dopaminergic Cell Line from Hydrogen Peroxide-Induced Cytotoxicity. Pharm. Biol..

[18] Abu-Khudir R., Ismail G.A., Diab T. (2021). Antimicrobial, Antioxidant, and Anti-Tumor Activities of Sargassum Linearifolium and Cystoseira Crinita from Egyptian Mediterranean Coast. Nutr. Cancer.

[19] Generalić Mekinić I., Čagalj M., Tabanelli G., Montanari C., Barbieri F., Skroza D., Šimat V. (2021). Seasonal Changes in Essential Oil Constituents of Cystoseira Compressa: First Report. Molecules.

[20] Oucif H., Adjout R., Sebahi R., Boukortt F.O., Ali-Mehidi S., El S.-M., Abi-Ayad A. (2017). Comparison of In Vitro Antioxidant Activity of Some Selected Seaweeds from Algerian West Coast. Afr. J. Biotechnol..

[21] Dulger G., Dulger B. (2014). Antibacterial Activity of Two Brown Algae (Cystoseira Compressa and Padina Pavonica) against Methicillin-Resistant Staphylococcus Aureus. Br. Microbiol. Res. J..

[22] Mhadhebi L., Dellai A., Clary-Laroche A., Said R.B., Robert J., Bouraoui A. (2012). Anti-Inflammatory and Antiproliferative Activities of C. Compressa. Drug Dev. Res..

[23] Kosanić M., Ranković B., Stanojković T. (2015). Biological Potential of Marine Macroalgae of the Genus Cystoseira. Acta Biol. Hung..

[24] Mhadhebi L., Mhadhebi A., Robert J., Bouraoui A. (2014). Antioxidant, Anti-Inflammatory and Antiproliferative Effects of Aqueous Extracts of Three Mediterranean Brown Seaweeds of the Genus Cystoseira. Iran. J. Pharm. Res..

[25] Hermund D.B. (2018). Antioxidant Properties of Seaweed-Derived Substances. Bioactive Seaweeds for Food Applications.

[26] Jacobsen C., Sørensen A.D.M., Holdt S.L., Akoh C.C., Hermund D.B. (2019). Source, Extraction, Characterization, and Applications of Novel Antioxidants from Seaweed. Annu. Rev. Food Sci. Technol..

[27] Polat S., Trif M., Rusu A., Šimat V., Čagalj M., Alak G., Meral R., Özogul Y., Polat A., Özogul F. (2021). Recent Advances in Industrial Applications of Seaweeds. Crit. Rev. Food Sci. Nutr..

[28] Maggio A., Alduina R., Oddo E., Piccionello A.P., Mannino A.M. (2020). Antibacterial Activity and HPLC Analysis of Extracts from Mediterranean Brown Algae. Plant Biosyst..

[29] Falace A., Zanelli E., Bressan G. (2005). Morphological and Reproductive Phenology of *Cystoseira Compressa* (Esper) Gerloff & Nizamuddin (Fucales, Fucophyceae) in the Gulf of Trieste (North Adriatic). Ann. Ser. Hist. Nat..

[30] Mannino A.M., Vaglica V., Cammarata M., Oddo E. (2016). Effects of Temperature on Total Phenolic Compounds in Cystoseira Amentacea (C. Agardh) Bory (Fucales, Phaeophyceae) from Southern Mediterranean Sea. Plant Biosyst.—Int. J. Deal. Asp. Plant Biol..

[31] Güner A., Köksal Ç., Erel Ş.B., Kayalar H., Nalbantsoy A., Sukatar A., Karabay Yavaşoğlu N.Ü. (2015). Antimicrobial and Antioxidant Activities with Acute Toxicity, Cytotoxicity and Mutagenicity of Cystoseira Compressa (Esper) Gerloff & Nizamuddin from the Coast of Urla (Izmir, Turkey). Cytotechnology.

[32] De La Fuente G., Fontana M., Asnaghi V., Chiantore M., Mirata S., Salis A., Damonte G., Scarfì S. (2021). The Remarkable Antioxidant and Anti-Inflammatory Potential of the Extracts of the Brown Alga Cystoseira Amentacea Var. Stricta. Mar. Drugs.

[33] Alghazeer R., Elmansori A., Sidati M., Gammoudi F., Azwai S., Naas H., Garbaj A., Eldaghayes I. (2017). In Vitro Antibacterial Activity of Flavonoid Extracts of Two Selected Libyan Algae against Multi-Drug Resistant Bacteria Isolated from Food Products. J. Biosci. Med..

[34] Abdeldjebbar F.Z., Bennabi F., Ayache A., Berrayah M., Tassadiat S. (2021). Synergistic Effect of Padina Pavonica and Cystoseira Compressa in Antibacterial Activity and Retention of Heavy Metals. Ukr. J. Ecol..

[35] Bouafif C., Messaoud C., Boussaid M., Langar H. (2018). Fatty Acid Profile of Cystoseira C. Agardh (Phaeophyceae, Fucales) Species from the Tunisian Coast: Taxonomic and Nutritional Assessments. Ciencias Mar..

[36] Zhong B., Robinson N.A., Warner R.D., Barrow C.J., Dunshea F.R., Suleria H.A.R. (2020). LC-ESI-QTOF-MS/MS Characterization of Seaweed Phenolics and Their Antioxidant Potential. Mar. Drugs.

[37] Jerković I., Cikoš A.-M., Babić S., Čižmek L., Bojanić K., Aladić K., Ul’yanovskii N.V., Kosyakov D.S., Lebedev A.T., Čož-Rakovac R. (2021). Bioprospecting of Less-Polar Constituents from Endemic Brown Macroalga Fucus Virsoides J. Agardh from the Adriatic Sea and Targeted Antioxidant Effects In Vitro and In Vivo (Zebrafish Model). Mar. Drugs.

[38] Ristivojević P., Jovanović V., Opsenica D.M., Park J., Rollinger J.M., Velicković T.Ć. (2021). Rapid Analytical Approach for Bioprofiling Compounds with Radical Scavenging and Antimicrobial Activities from Seaweeds. Food Chem..

[39] Das U.N. (2018). Arachidonic Acid and Other Unsaturated Fatty Acids and Some of Their Metabolites Function as Endogenous Antimicrobial Molecules: A Review. J. Adv. Res..

[40] Chanda W., Joseph T.P., Guo X.F., Wang W.D., Liu M., Vuai M.S., Padhiar A.A., Zhong M.T. (2018). Effectiveness of Omega-3 Polyunsaturated Fatty Acids against Microbial Pathogens. J. Zhejiang Univ. Sci. B.

[41] Shin S.Y., Bajpai V.K., Kim H.R., Kang S.C. (2007). Antibacterial Activity of Bioconverted Eicosapentaenoic (EPA) and Docosahexaenoic Acid (DHA) against Foodborne Pathogenic Bacteria. Int. J. Food Microbiol..

[42] Cvitković D., Dragović-Uzelac V., Dobrinčić A., Čož-Rakovac R., Balbino S. (2021). The Effect of Solvent and Extraction Method on the Recovery of Lipid Fraction from Adriatic Sea Macroalgae. Algal Res..

[43] Lacey R.W., Lord V.L. (1981). Sensitivity of Staphylococci to Fatty Acids: Novel Inactivation of Linolenic Acid by Serum. J. Med. Microbiol..

[44] Le P., Desbois A. (2017). Antibacterial Effect of Eicosapentaenoic Acid against Bacillus Cereus and Staphylococcus Aureus: Killing Kinetics, Selection for Resistance, and Potential Cellular Target. Mar. Drugs.

[45] Chen C.-H., Wang Y., Nakatsuji T., Liu Y.-T., Zouboulis C.C., Gallo R.L., Zhang L., Hsieh M.-F., Huang C.-M. (2011). An Innate Bactericidal Oleic Acid Effective against Skin Infection of Methicillin-Resistant Staphylococcus Aureus: A Therapy Concordant with Evolutionary Medicine. J. Microbiol. Biotechnol..

[46] Čagalj M., Skroza D., Tabanelli G., Özogul F., Šimat V. (2021). Maximizing the Antioxidant Capacity of Padina Pavonica by Choosing the Right Drying and Extraction Methods. Processes.

[47] Amerine M.A., Ough C.S. (1980). Methods for Analysis of Musts and Wines.

[48] Benzie I.F.F., Strain J.J. (1996). The Ferric Reducing Ability of Plasma (FRAP) as a Measure of “Antioxidant Power”: The FRAP Assay. Anal. Biochem..

[49] Milat A.M., Boban M., Teissedre P.L., Šešelja-Perišin A., Jurić D., Skroza D., Generalić-Mekinić I., Ljubenkov I., Volarević J., Rasines-Perea Z. (2019). Effects of Oxidation and Browning of Macerated White Wine on Its Antioxidant and Direct Vasodilatory Activity. J. Funct. Foods.

[50] Prior R.L., Hoang H., Gu L., Wu X., Bacchiocca M., Howard L., Hampsch-Woodill M., Huang D., Ou B., Jacob R. (2003). Assays for Hydrophilic and Lipophilic Antioxidant Capacity (Oxygen Radical Absorbance Capacity (ORACFL)) of Plasma and Other Biological and Food Samples. J. Agric. Food Chem..

[51] Burčul F., Generalić Mekinić I., Radan M., Rollin P., Blažević I. (2018). Isothiocyanates: Cholinesterase Inhibiting, Antioxidant, and Anti-Inflammatory Activity. J. Enzym. Inhib. Med. Chem..

[52] Skroza D., Šimat V., Smole Možina S., Katalinić V., Boban N., Generalić Mekinić I. (2019). Interactions of Resveratrol with Other Phenolics and Activity against Food-Borne Pathogens. Food Sci. Nutr..

[53] Elez Garofulić I., Malin V., Repajić M., Zorić Z., Pedisić S., Sterniša M., Smole Možina S., Dragović-Uzelac V. (2021). Phenolic Profile, Antioxidant Capacity and Antimicrobial Activity of Nettle Leaves Extracts Obtained by Advanced Extraction Techniques. Molecules.

[54] Verni M., Pontonio E., Krona A., Jacob S., Pinto D., Rinaldi F., Verardo V., Díaz-de-Cerio E., Coda R., Rizzello C.G. (2020). Bioprocessing of Brewers’ Spent Grain Enhances Its Antioxidant Activity: Characterization of Phenolic Compounds and Bioactive Peptides. Front. Microbiol..

[55] Šimat V., Vlahović J., Soldo B., Generalić Mekinić I., Čagalj M., Hamed I., Skroza D. (2020). Production and Characterization of Crude Oils from Seafood Processing by-Products. Food Biosci..

